# Clinical symptoms and related risk factors in pulmonary embolism patients and cluster analysis based on these symptoms

**DOI:** 10.1038/s41598-017-14888-7

**Published:** 2017-11-02

**Authors:** Qiao-ying Ji, Mao-feng Wang, Cai-min Su, Qiong-fang Yang, Lan-fang Feng, Lan-yan Zhao, Shuang-yan Fang, Fen-hua Zhao, Wei-min Li

**Affiliations:** 1 Department of Respiratory, Affiliated Dongyang Hospital of Wenzhou Medical University, Dongyang, Zhejiang, 322100 China; 2 Department of Biomedical Sciences Laboratory, Affiliated Dongyang Hospital of Wenzhou Medical University, Dongyang, Zhejiang 322100 China; 3 Department of Radiology, Affiliated Dongyang Hospital of Wenzhou Medical University, Dongyang, Zhejiang, 322100 China; 4 Department of Cardiology, Affiliated Dongyang Hospital of Wenzhou Medical University, Dongyang, Zhejiang, 322100 China

## Abstract

Pulmonary embolism (PE) remains largely underdiagnosed due to nonspecific symptoms. This study aims to evaluate typical symptoms of PE patients, their related predictors, and to differentiate typical clusters of patients and principal components of PE symptoms. Clinical data from a total of 551 PE patients between January 2012 and April 2016 were retrospectively reviewed. PE was diagnosed according to the European Society of Cardiology Guidelines. Logistic regression models, system clustering method, and principal component analysis were used to identify potential risk factors, different clusters of the patients, and principal components of PE symptoms. The most common symptoms of PE were dyspnea, cough, and tachypnea in more than 60% of patients. Some combined chronic conditions, laboratory and clinical indicators were found to be related to these clinical symptoms. Our study also suggested that PE is associated with a broad list of symptoms and some PE patients might share similar symptoms, and some PE symptoms were usually cooccurrence. Based on ten symptoms generated from our sample, we classified the patients into five clusters which represent five groups of PE patients during clinical practice, and identified four principal components of PE symptoms. These findings will improve our understanding of clinical symptoms and their potential combinations which are helpful for clinical diagnosis of PE.

## Introduction

Pulmonary embolism (PE) is a potentially life-threatening disease with significant morbidity and potentially fatal outcomes^1,2^. The prognosis of PE relies on timely and accurate diagnosis, reasonable risk stratification, and well-monitored anticoagulation^3^. However, PE remains largely underdiagnosed due to the vague and nonspecific clinical manifestations^4–6^. Prompt diagnosis and recognition of PE patients has been shown to reduce mortality and morbidity^7^.

The clinical presentation of PE is very nonspecific and related to a broad list of symptoms^8^. In fact, the classic clinical symptoms (e.g. the triad syndrome including hemoptysis, dyspnea, and pleuritic pain) were not common and not easily recognized during current clinical practice^6^. The overall presentation could be easily confused with systemic disorders or other cardio-pulmonary diseases^9^. It was also important to realize that PE patients might be asymptomatic which further underdiagnosed this disease^10^.

In the process of diagnosis, clinical symptoms were recommended to be firstly assessed ideally by a validated prediction model^11^, although final diagnosis should be mainly based on clinical findings, laboratory tests, and imaging data. Previous studies demonstrated that dyspnea followed by pleuritic pain and cough were the most common symptoms of PE^12^. Surprisingly, a large amount of patients, including those with massive PE, had mild or nonspecific symptoms or even were asymptomatic^13^. For instance, one systematic review including 28 studies revealed that one-third of 5,233 patients with a deep vein thrombosis (DVT) had asymptomatic PE^14^. Moreover, some patients had a delayed presentation of related symptoms over weeks or days^15^. Therefore, well understanding of typical clinical symptoms associated with PE, their potential predictors, and potential co-occurrence and combination of these symptoms would improve diagnosis and prognosis of PE. However, few studies had focused on this topic and no studies were found to cluster PE symptoms and patients to date. Few related studies were all based on Western populations^6,16^, and Chinese patients might have different characteristics due to racial differences^17,18^.

Based on a large sample of PE patients in a comprehensive hospital in China, this study aims to assess typical clinical symptoms, to identify related clinical and laboratory indicators of these symptoms, to group the patients into different clusters based on the presentations of symptoms, and to identify the principal components of these symptoms. This study will improve our understanding of clinical symptoms and their potential combinations which are helpful for clinical diagnosis of PE.

## Methods

### Ethics statement

This study was approved by the Medical Ethics Committee of Affiliated Dongyang Hospital of Wenzhou Medical University (Dongyang, China). Informed consent was obtained from all enrolled patients. Patients records/information was anonymized and de-identified prior to analysis. All experiments were performed in accordance with relevant guidelines and regulations.

### Subjects and sample collection

Clinical data from a total of 551 patients hospitalized at the Affiliated Dongyang Hospital of Wenzhou Medical University between January 2012 and April 2016 were retrospectively reviewed. According to the criteria of European Society of Cardiology Guidelines, PE was confirmed by an identified filling defect in the pulmonary artery system in a CT pulmonary angiography (CTPA) or a positive venous ultrasound of extremity deep venous thrombosis (DVT) in patients with typical symptoms of PE (Pleuritic pain or dyspnea). In addition, a positive D-dimmer or ventilation-perfusion (V/Q) scintigraphy supports a high probability for PE, as recommended in those guidelines. All clinical data including symptoms, vital signs, comorbidities, and laboratory indicators were collected on admission. Ten symptoms were included in the analysis including dyspnea, tachypnea, hemoptysis, pleuritic pain, hydrothorax, syncope, hypoxia, fever, cough, and edema in lower extremities. Concurrent chest radiographs, CT scan and echocardiographic examination were performed and recorded.

### CTPA examination

The thoracic CT scans were performed using a 64-detector multi-sectional CT scanner (Brilliance 64-slice; PHILIPS, Amsterdam, Netherlands) with an intravenously injected contrast agent. Scanned with multi-slice spiral CT, collimation of 0.6, rotation time of 0.5 s, slice thickness of 5 mm, and pitch of 1.0, contrast agent (100 ml) was injected at 4 ml/s. CTPA results were categorized as positive for PE if an intraluminal filling defect was observed within a pulmonary arterial vessel and were considered negative if no filling defect was seen. Scans were considered technically inadequate only if main or lobar pulmonary vessels were not visualized.

### Statistical Analysis

Characteristics were presented as mean ± standard deviation (SD) for continuous variables and percentage for categorical variables. Multivariate logistic regression models were used to identify the associations between clinical indicators/predictors and PE symptoms. System clustering method was used to identify typical clusters of PE patients based on the symptoms. In this method, Euclidean distance was used to measure similarity of objects in the symptoms of PE Patients, and Ward’s method was performed to group objects to clusters. Four cluster evaluation statistics including R-squared, semi-partial R-squared, Pseudo F and Pseudo T-squared were plotted in the hierarchical analysis to determine the optimal number of clusters. According to the plots, we usually defined 4–6 clusters. Within this range of number of clusters, we chose the number with smaller semi-partial R-squared, larger R-squared, larger Pseudo F, and smaller Pseudo T-squared statistics. Chi-square Fisher’s exact test was utilized to determine whether cluster membership was significantly associated with different clusters. To determine the extent to which the clusters based on presentations of PE correspond to differences in other indicators, a series of one-way ANOVA tests were performed for other indicators with different clusters. Principal component analysis (PCA) was used to identify potential principal components of PE symptoms. All principal components with eigenvalue greater than 1 were remained. All significance levels were set at 0.05. Statistical analyses were run in SAS 9.4.

## Results

### Characteristics of the study population

A total of 551 patients with PE were included in this study. Table 1 presented the characteristics of the study population. Patients presented a broad range of clinical symptoms. Among all symptoms, dyspnea was the most common presenting symptom in 64.1% of cases with PE, followed by cough (60.4%), tachypnea (60.4%) and hypoxia (57.9%), while the other symptoms included hydrothorax, edema in lower extremities, fever, syncope, hemoptysis and pleuritic pain, accounting for 26.9%, 23.6%, 22.1%, 12.7%, 11.8% and 8.2%, respectively. PE patients were usually combined with hypertension (45.2%) and chronic obstructive pulmonary disease (COPD) (32.1%).

**Table 1:** Demographic and clinical characteristics of study subjects (N = 551).

**Variables**	**Mean (SD) or N (%)**
Age	71.3 ± 12.2
Male	299 (54.26)
*Presentation of symptoms*	
Dyspnea	353 (64.07)
Tachypnea	333 (60.44)
Hemoptysis	65 (11.8)
Pleuritic pain	45 (8.17)
Hydrothorax	148 (26.86)
Syncope	70 (12.7)
Hypoxia	319 (57.89)
Fever	122 (22.14)
Cough	333 (60.44)
Edema in lower extremities (Class 1–3)	130 (23.59)
*Comorbidity*	
Hypertension	249 (45.19)
AF	72 (13.07)
CHD	88 (15.97)
DM	64 (11.62)
COPD	177 (32.12)
Thrombosis in lower extremities	124 (22.5)
Cancers	69 (12.52)
*Laboratory indicators*	
WBC	7.86 ± 3.64
NGC	0.72 ± 0.18
RBC	4.04 ± 0.66
HGB	122.03 ± 19.89
PLT	206.04 ± 75.34
ALB	36.35 ± 4.51
D_D	5.51 ± 5.79
*Blood gas indicators*	
PH	7.43 ± 0.06
PPO	85.99 ± 32.15
PPOCD	40.7 ± 12.97
LAC	2.07 ± 5.84
*Ultrasonic indicators*	
PAP (Class 1–3)	340 (61.71)
TTVR (Class 1–3)	196 (35.57)

Abbreviations: AF: atrial fibrillation; CHD: coronary heart disease: DM: diabetes mellitus; COPD: chronic obstructive pulmonary disease; WBC, white blood cell; NGC, neutrophilic granulocyte, RBC, red blood cell; HGB, hemoglobin; PLT, platelet; ALB, Albumin; D_D, D-dimer; PPO, arterial oxygen tension; PPOCD, arterial carbon dioxide tension; LAC, lactic acid; PAP, pulmonary artery pressure; TTVR, Three tip valve regurgitation.

### Associations between comorbidities and symptoms

The results of associations between comorbidities and each symptom were shown in Table 2. The PE patients with atrial fibrillation (AF) were more likely to present tachypnea, hemoptysis, and hydrothorax compared to those without AF. The patients with coronary heart disease (CHD) experienced higher odds of presenting dyspnea and tachypnea, and those with CDPD experienced higher odds of presenting dyspnea, tachypnea, hypoxia, and cough. The patients with lower extremities thrombosis were more likely to present edema in lower extremities. However, the PE patients with hypertension or diabetes, COPD, and lower extremities thrombosis experienced lower odds of presenting cough, syncope, and dyspnea, respectively.

**Table 2:** The associations between comorbidities and presentation of symptoms from logistic regression models (N = 551)

	OR (95 CI)						
	Hypertension	AF	CHD	DM	COPD	Thrombosis in lower extremities	Cancers
Dyspnea	0.81 (0.56–1.17)	1.61 (0.91–2.88)	**2.70 (1.50–4.83)**	0.86 (0.50–1.48)	**6.79 (4.07–11.34)**	**0.57 (0.38–0.86)**	0.83 (0.49–1.39)
Tachypnea	0.79 (0.55–1.14)	**1.87 (1.04–3.35)**	**2.64 (1.50–4.65)**	0.95 (0.56–1.63)	**9.12 (5.37–15.49)**	0.72 (0.48–1.08)	0.83 (0.50–1.40)
Hemoptysis	0.75 (0.43–1.32)	**2.46 (1.22–4.93)**	1.05 (0.50–2.21)	0.63 (0.24–1.66)	1.72 (0.98–3.04)	0.83 (0.43–1.62)	0.71 (0.31–1.65)
Pleuritic pain	0.58 (0.30–1.14)	0.69 (0.24–2.05)	0.88 (0.35–2.20)	0.34 (0.08–1.45)	0.75 (0.37–1.53)	0.86 (0.40–1.85)	0.78 (0.30–2.07)
Hydrothorax	1.24 (0.84–1.83)	**3.05 (1.80–5.15)**	1.19 (0.72–1.98)	0.73 (0.39–1.38)	1.40 (0.93–2.10)	1.11 (0.71–1.75)	0.94 (0.52–1.69)
Syncope	1.13 (0.67–1.90)	0.74 (0.32–1.71)	0.78 (0.36–1.66)	0.68 (0.28–1.64)	**0.25 (0.12–0.54)**	0.61 (0.32–1.19)	0.62 (0.25–1.49)
Hypoxia	1.38 (0.96–1.97)	1.08 (0.64–1.84)	0.86 (0.54–1.39)	1.36 (0.79–2.35)	**2.11 (1.42–3.14)**	0.79 (0.53–1.19)	0.66 (0.40–1.11)
Fever	0.76 (0.50–1.16)	0.91 (0.49–1.70)	0.67 (0.37–1.23)	0.47 (0.22–1.01)	1.28 (0.82–1.99)	1.31 (0.82–2.09)	0.63 (0.32–1.24)
Cough	0.58 (0.40–0.83)	1.15 (0.66–1.99)	0.83 (0.51–1.35)	**0.53 (0.31–0.91)**	**11.49 (6.45–20.47)**	0.72 (0.47–1.09)	1.14 (0.66–1.96)
Edema in lower extremities	1.06 (0.71–1.59)	1.10 (0.62–1.97)	1.40 (0.83–2.34)	1.20 (0.67–2.16)	1.04 (0.67–1.61)	**3.12 (2.02–4.80)**	1.09 (0.60–1.97)

Abbreviations: OR, odds ratio; CI, confidence interval; AF: atrial fibrillation; CHD: coronary heart disease: DM: diabetes mellitus; COPD: chronic obstructive pulmonary disease.

Age and sex were adjusted in the models.

Bold: p < 0.05.

### Associations between laboratory indicators and symptoms

The results of associations between some laboratory indicators and each symptom were summarized in Table 3. The counts of white blood cell (WBC), neutrophilic granulocyte, red blood cell (RBC), and D-dimer (D-D) were usually associated with increased odds of presenting these symptoms. For example, the count of WBC was positively associated with presenting of dyspnea, tachypnea, hydrothorax, syncope, hypoxia, and fever. The level of albumin (ALB) was usually associated with decreased odds of presenting symptoms including dyspnea, tachypnea, hydrothorax, fever, and cough. Hemoglobin and platelet were occasionally associated with some symptoms. Arterial carbon dioxide tension (PPOCD) and three tip valve regurgitation (TTVR) were associated with increased odds of presenting several symptoms, while the PH value was associated with decreased odds of presenting dyspnea, tachypnea, hydrothorax, and syncope (Table 4).

**Table 3:** The associations between laboratory indicators and presentation of symptoms from logistic regression models (N = 551)

	OR (95 CI)						
	WBC	NGC	RBC	HGB	PLT	ALB	D_D
Dyspnea	**1.06 (1.01–1.12)**	**12.95 (2.97–56.56)**	1.11 (0.85–1.45)	1.00 (0.99–1.01)	1.00 (0.99–1.00)	**0.95 (0.91–0.98)**	0.98 (0.95–1.01)
Tachypnea	**1.07 (1.02–1.13)**	**8.57 (2.96–24.86)**	1.04 (0.80–1.36)	1.00 (0.99–1.01)	1.00 (0.99–1.00)	**0.93 (0.89–0.96)**	0.98 (0.95–1.01)
Hemoptysis	0.93 (0.86–1.02)	0.40 (0.05–3.32)	1.02 (0.69–1.52)	1.00 (0.98–1.01)	1.00 (0.99–1.00)	0.99 (0.93–1.05)	0.97 (0.92–1.02)
Pleuritic pain	1.04 (0.96–1.12)	7.67 (0.87–67.33)	0.9 (0.57–1.43)	1.00 (0.98–1.01)	1.00 (0.99–1.00)	0.98 (0.92–1.05)	0.99 (0.94–1.04)
Hydrothorax	**1.07 (1.02–1.13)**	3.32 (0.85–12.89)	0.84 (0.63–1.13)	0.99 (0.98–1.00)	1.00 (0.99–1.00)	0.89 (0.85–0.93)	1.05 (1.02–1.08)
Syncope	**1.07 (1.00–1.13)**	0.66 (0.12–3.71)	0.91 (0.62–1.34)	1.00 (0.99–1.02)	1.00 (0.99–1.00)	0.99 (0.94–1.05)	1.01 (0.97–1.05)
Hypoxia	**1.06 (1.01–1.11)**	1.20 (0.45–3.21)	**1.76 (1.33–2.32)**	**1.01 (1.01–1.02)**	1.00 (0.99–1.00)	0.98 (0.94–1.01)	1.01 (0.98–1.04)
Fever	**1.13 (1.07–1.19)**	**62.35 (9.97–389.77)**	**0.67 (0.49–0.92)**	**0.99 (0.98–1.00)**	1.00 (0.99–1.00)	**0.91 (0.87–0.95)**	**1.04 (1.01–1.07)**
Cough	1.02 (0.97–1.07)	1.81 (0.59–5.55)	0.96 (0.73–1.27)	0.99 (0.99–1.00)	**1.00 (1.00–1.01)**	**0.93 (0.89–0.97)**	**0.97 (0.94–0.99)**
Edema in lower extremities	0.97 (0.92–1.03)	0.54 (0.13–2.20)	**1.37 (1.01–1.87)**	1.01 (0.99–1.02)	1.00 (0.99–1.00)	1.00 (0.96–1.05)	1.01 (0.98–1.04)

Abbreviations: OR, odds ratio; CI, confidence interval; WBC, white blood cell; NGC, neutrophilic granulocyte, RBC, red blood cell; HGB, hemoglobin; PLT, platelet; ALB, Albumin; D_D, D–dimer.

Age and sex were adjusted in the models.

Bold: p < 0.05.

**Table 4:** The associations between blood gas/ultrasonic indicators and presentation of symptoms from logistic regression models (N = 551)

	OR (95 CI)					
	PH	PPO	PPOCD	LAC	PAP	TTVR
Dyspnea	**0.02 (0.00–0.47)**	0.99 (0.99–1.00)	**1.04 (1.02–1.06)**	1.01 (0.97–1.04)	0.93 (0.78–1.11)	**1.63 (1.28–2.07)**
Tachypnea	**0.01 (0.00–0.20)**	1.00 (0.99–1.00)	**1.04 (1.02–1.06)**	1.01 (0.98–1.04)	1.00 (0.84–1.20)	**1.61 (1.27–2.03)**
Hemoptysis	0.35 (0.01–16.29)	1.00 (0.99–1.01)	1.00 (0.99–1.02)	0.94 (0.84–1.07)	0.96 (0.73–1.26)	1.25 (0.91–1.70)
Pleuritic pain	0.46 (0.01–38.43)	1.00 (0.99–1.01)	1.00 (0.98–1.02)	0.90 (0.78–1.04)	1.09 (0.81–1.48)	0.91 (0.61–1.35)
Hydrothorax	**0.05 (0.00–0.86)**	1.00 (0.99–1.00)	**1.02 (1.00–1.03)**	1.01 (0.98–1.04)	0.90 (0.73–1.10)	**1.88 (1.48–2.39)**
Syncope	**0.01 (0.00–0.16)**	**1.01 (1.00–1.02)**	0.99 (0.96–1.01)	1.01 (0.98–1.05)	0.87 (0.66–1.15)	**0.67 (0.47–0.95)**
Hypoxia	0.16 (0.01–2.51)	**0.95 (0.94–0.96)**	**1.04 (1.02–1.06)**	1.04 (0.98–1.10)	**1.25 (1.04–1.50)**	1.13 (0.91–1.40)
Fever	5.91 (0.18–197.85)	1.00 (0.99–1.01)	0.99 (0.97–1.01)	0.99 (0.95–1.04)	1.01 (0.82–1.25)	**0.72 (0.55–0.94)**
Cough	0.32 (0.02–5.1)	1.00 (0.99–1.00)	**1.05 (1.03–1.06)**	1.00 (0.97–1.03)	0.92 (0.76–1.10)	1.19 (0.95–1.49)
Edema in lower extremities	0.14 (0.01–2.18)	1.00 (0.99–1.00)	1.01 (0.99–1.02)	0.99 (0.95–1.03)	0.96 (0.78–1.17)	**1.43 (1.13–1.81)**

Abbreviations: OR, odds ratio; CI, confidence interval; PPO, arterial oxygen tension; PPOCD, arterial carbon dioxide tension; LAC, lactic acid; PAP, pulmonary artery pressure; TTVR, Three tip valve regurgitation.

Age and sex were adjusted in the models.

Bold: p < 0.05.

### Five clusters of patients from cluster analysis

**Figure 1:**
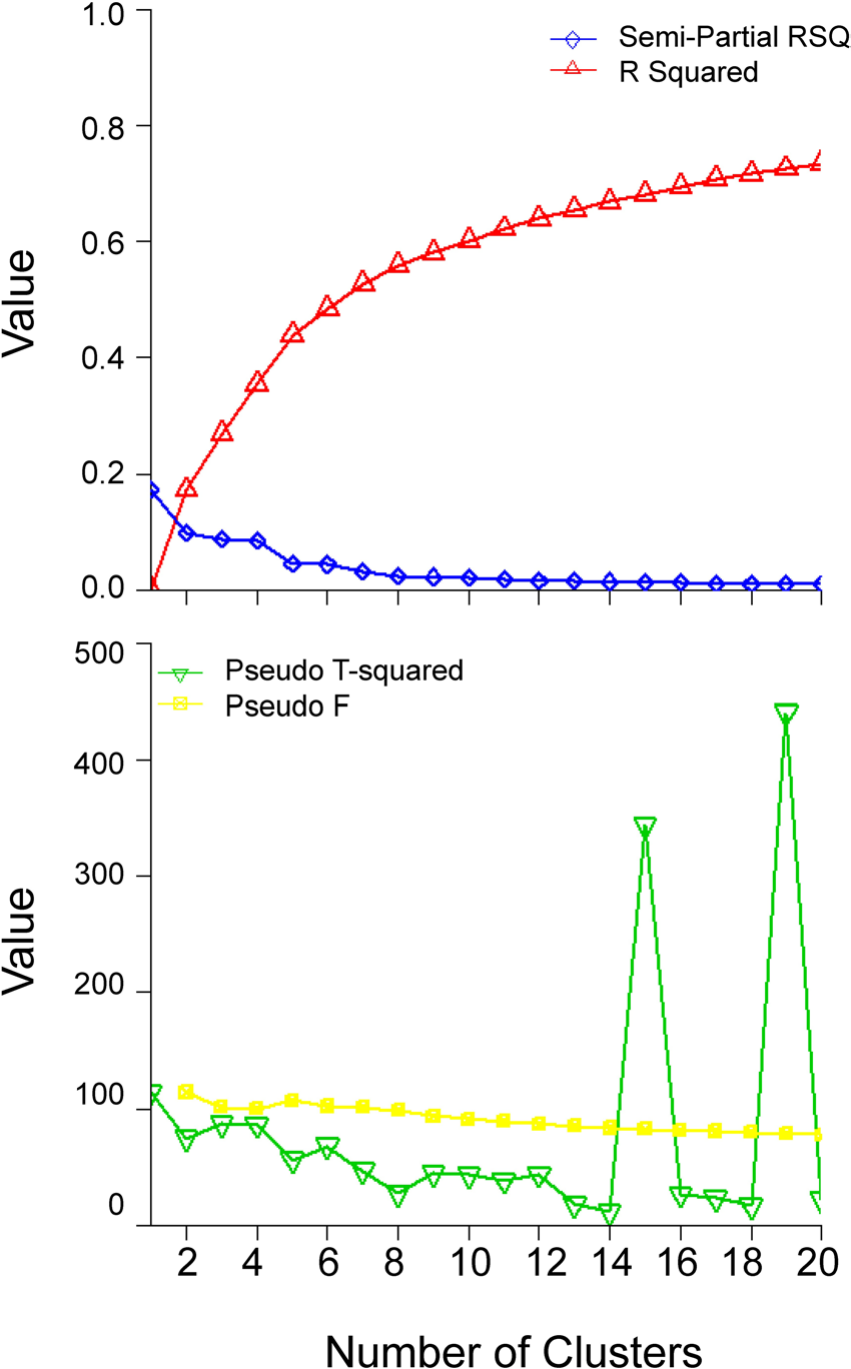
Evaluation of cluster analysis results.

Based on ten main symptoms presented among our PE patient sample, we generated five clusters of patients according to cluster analysis approach. We evaluated the cluster analysis by R-squared, semi-partial R-squared, Pseudo F and Pseudo T-squared statistics, and the plots results suggested noticeable improvement at around five clusters. Thus, five is the most optimal number, where both the semi-partial R-squared and Pseudo T-squared were relatively small, and both R-squared and Pseudo F were relatively large (Figure 1). The cluster history results were presented in Table 1S in the supplementary file. Table 5 showed the number and proportion of each symptom in each of five clusters. Almost half of the patients (N = 250) were classified into Cluster 3, and a quarter of those (N = 136) were classified into Cluster 2. Among patients in Cluster 3, almost all presented dyspnea (98.8%) and tachypnea (92.8%), and most of them presented hypoxia (70.8%) and cough (71.6%). For Cluster 2, we could find that the patients have no specific/typical symptoms. Among patients in Cluster 1, all presented syncope, and almost half of those presented dyspnea, hypoxia, tachypnea, and cough, but few presented pleuritic pain, hemoptysis, and edema in lower extremities. Table 6 summarized age, gender, comorbidities, and clinical indicators related to PE by five clusters. For example, in Cluster 3, the patients were the oldest, were most likely to have COPD, had the lowest arterial oxygen tension (PPO), the highest PPOCD, and pulmonary artery pressure (PAP).

**Table 5:** Prevalence of symptoms by the clusters of patients with pulmonary embolism (N = 551).

	Cluster1 (N = 65)	Cluster2 (N = 136)	Cluster3 (N = 250)	Cluster4 (N = 56)	Cluster5 (N = 44)	P–value
Dyspnea	29 (44.6%)	2 (1.5%)	247 (98.8%)	41 (73.2%)	34 (77.3%)	<0.001
Tachypnea	24 (36.9%)	4 (2.9%)	232 (92.8%)	42 (75.0%)	31 (70.5%)	<0.001
Hemoptysis	2 (3.1%)	0 (0.0%)	0 (0.0%)	56 (100.0%)	7 (15.9%)	<0.001
Pleuritic pain	1 (1.5%)	0 (0.0%)	0 (0.0%)	0 (0.0%)	44 (100.0%)	<0.001
Hydrothorax	10 (15.4%)	12 (8.8%)	89 (35.6%)	20 (35.7%)	17 (38.6%)	<0.001
Syncope	65 (100.0%)	1 (0.7%)	1 (0.4%)	0 (0.0%)	3 (6.8%)	<0.001
Hypoxia	31 (47.7%)	54 (39.7%)	177 (70.8%)	32 (57.1%)	25 (56.8%)	<0.001
Fever	11 (16.9%)	34 (25.0%)	54 (21.6%)	10 (17.9%)	13 (29.5%)	0.457
Cough	24 (36.9%)	46 (33.8%)	179 (71.6%)	53 (94.6%)	31 (70.5%)	<0.001
Edema in lower extremities	5 (7.7%)	40 (29.4%)	70 (28.0%)	10 (17.9%)	5 (11.4%)	0.026

**Table 6:** Prevalence of symptoms by the clusters of patients with pulmonary embolism (N = 551)

	Cluster1 (N = 65)	Cluster2 (N = 136)	Cluster3 (N = 250)	Cluster4 (N = 56)	Cluster5 (N = 44)	P–value
Age	70.87 ± 12.33	69.48 ± 12.34	73.11 ± 11.61	69.57 ± 10.26	69.55 ± 15.7	0.030
Male	33 (50.8%)	64 (47.1%)	131 (52.4%)	42 (75.0%)	29 (65.9%)	0.004
Comorbidity						
Hypertension	32 (49.2%)	67 (49.3%)	116 (46.4%)	20 (35.7%)	14 (31.8%)	0.153
AF	7 (10.8%)	12 (8.8%)	37 (14.8%)	12 (21.4%)	4 (9.1%)	0.127
CHD	9 (13.8%)	13 (9.6%)	50 (20.0%)	10 (17.9%)	6 (13.6%)	0.102
DM	6 (9.2%)	19 (14.0%)	33 (13.2%)	4 (7.1%)	2 (4.5%)	0.209
COPD	7 (10.8%)	13 (9.6%)	119 (47.6%)	26 (46.4%)	12 (27.3%)	<0.001
Thrombosis in lower extremities	10 (15.4%)	49 (36.0%)	48 (19.2%)	9 (16.1%)	8 (18.2%)	0.001
Cancers	6 (9.2%)	22 (16.2%)	31 (12.4%)	5 (8.9%)	5 (11.4%)	0.555
Laboratory indicators						
WBC	8.53 ± 4.35	7.55 ± 3.77	7.93 ± 3.46	7.17 ± 2.89	8.39 ± 3.86	0.185
NGC	0.71 ± 0.13	0.69 ± 0.12	0.73 ± 0.12	0.7 ± 0.12	0.81 ± 0.47	0.002
RBC	3.97 ± 0.63	4.02 ± 0.62	4.05 ± 0.66	4.1 ± 0.71	4.04 ± 0.75	0.886
HGB	122.34 ± 18.37	121.77 ± 19.7	121.76 ± 19.97	122.59 ± 20.78	123.2 ± 21.78	0.991
PLT	192.89 ± 62.08	215.67 ± 86.74	204.43 ± 72.74	202.27 ± 61.68	209.61 ± 84.45	0.337
ALB	36.21 ± 4.16	37.38 ± 4.57	35.85 ± 4.64	36.33 ± 3.64	36.22 ± 4.68	0.035
D_D	5.45 ± 5.74	6.83 ± 6.31	5.2 ± 5.33	4.4 ± 6.18	4.71 ± 5.72	0.029
Blood gas indicators						
PH	7.4 ± 0.1	7.44 ± 0.06	7.43 ± 0.06	7.42 ± 0.04	7.42 ± 0.05	0.001
PPO	96.67 ± 44.34	89.03 ± 35.13	80.14 ± 26.72	90.78 ± 30.13	87.99 ± 26.3	0.001
PPOCD	39.14 ± 7.56	37.51 ± 10.47	42.46 ± 13.19	42.18 ± 11.46	40.96 ± 22.13	0.005
LAC	2.71 ± 3.05	2.05 ± 7.94	2.16 ± 6.15	1.55 ± 0.71	1.29 ± 1.47	0.723
Ultrasonic indicators						
PAP (Class 1–3)	32 (49.2%)	74 (54.4%)	166 (66.4%)	41 (73.2%)	27 (61.4%)	0.013
TTVR (Class 1–3)	19 (29.2%)	50 (36.8%)	91 (36.4%)	17 (30.4%)	19 (43.2%)	0.927

Abbreviations: AF: atrial fibrillation; CHD: coronary heart disease: DM: diabetes mellitus; COPD: chronic obstructive pulmonary disease; WBC, white blood cell; NGC, neutrophilic granulocyte, RBC, red blood cell; HGB, hemoglobin; PLT, platelet; ALB, Albumin; D_D, D-dimer; PPO, arterial oxygen tension; PPOCD, arterial carbon dioxide tension; LAC, lactic acid; PAP, pulmonary artery pressure; TTVR, Three tip valve regurgitation.

### PCA of PE symptoms

**Figure 2:**
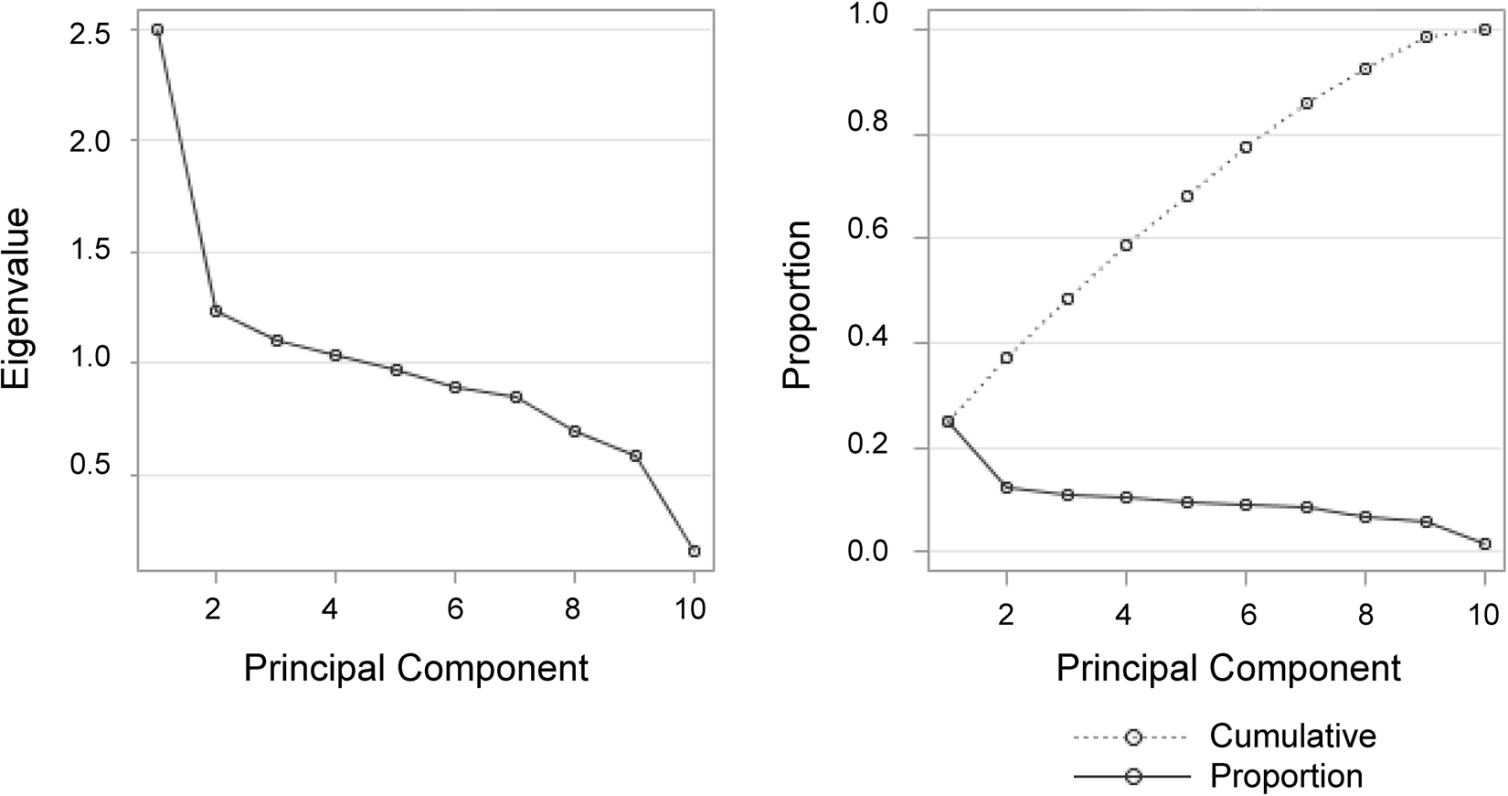
The graph results of PCA.

The principal components with eigenvalue greater than 1 were retained to account for as large as possible proportion of the total variability in the component measures. The eigenvalues of the correlation matrix from PCA were shown in Table 1S, and the graph results of PCA were shown in Fig. 2. The loadings of ten symptoms for all principal components were presented in Table 7. In the first principal component which accounted for 25% of the variance, the symptoms with large loadings included tachypnea, dyspnea, cough, hydrothorax and hypoxia.

**Table 7:** Eigenvectors of ten principal components from PCA

	**PC1**	**PC2**	**PC3**	**PC4**	**PC5**	**PC6**	**PC7**	**PC8**	**PC9**	**PC10**
Dyspnea	0.526	−0.242	0.170	−0.199	0.066	−0.093	0.202	−0.034	−0.280	0.680
Tachypnea	0.552	−0.194	0.127	−0.170	0.000	−0.113	0.213	0.004	−0.168	−0.726
Hemoptysis	0.159	0.407	−0.524	−0.276	−0.252	0.417	−0.029	0.234	−0.407	0.007
Pleuritic pain	0.102	0.359	0.057	−0.144	0.869	0.036	−0.075	0.262	0.078	−0.019
Hydrothorax	0.308	0.040	−0.081	0.443	0.179	0.535	−0.058	−0.613	0.053	−0.010
Syncope	−0.203	0.056	0.531	−0.130	−0.097	0.581	0.518	0.176	0.102	−0.007
Hypoxia	0.278	−0.067	0.394	0.162	−0.201	0.225	−0.689	0.409	0.063	0.011
Fever	0.090	0.444	0.160	0.678	−0.093	−0.281	0.280	0.217	−0.310	0.017
Cough	0.401	0.309	−0.159	−0.051	−0.243	−0.117	0.181	0.081	0.771	0.100
Edema in lower extremities	0.022	−0.554	−0.428	0.365	0.166	0.189	0.225	0.498	0.120	0.016

Abbreviations: PCA: principal component analysis; PC: principal component.

## Discussion

In our study, the most common symptoms of PE were dyspnea, cough, and tachypnea in more than 60% of patients. Some combined chronic conditions, and laboratory and clinical indicators were found to be related to these clinical symptoms among PE patients. The present study also suggested that PE is associated with a broad list of symptoms and some PE patients might share similar symptoms. Based on ten symptoms generated from our sample, we classified the patients into five clusters which represent five groups of PE patients during clinical practice. Four principal components of symptoms were identified and the tachypnea, dyspnea, cough, hydrothorax, and hypoxia were the most common symptoms in the largest principal component which accounted for 25% of variability of the PE symptoms.

The diagnosis of PE remains challenging, particularly due to the absence of commonly associated symptoms and signs in this disease. The majority of well-known symptoms in our sample are similar in prevalence to those described in prior studies^2,5,6,18–23^. However, the results are still inconsistent across previous studies. For example, some studies showed that pleuritic pain and hemoptysis were the most frequent mode of presentation in PE patients^2^. A recent study indicated that most PE patients featured at least one of the four following symptoms: sudden onset dyspnea, pleuritic pain, syncope, and hemoptysis^11^. In the present study, however, fewer PE patients had pleuritic pain and hemoptysis. Hemoptysis has been traditionally taught as a classically described symptom in the presentation of PE^12,20,24–27^. Previous studies have reported that the occurrence of hemoptysis in PE is to be as high as 20–25%^12^. However, in our study, hemoptysis was noticed only in 11.8% of PE patients. We hypothesize that the decrease in the incidence of hemoptysis might be related to the wide availability of CT scans, allowing of early detection and timely anticoagulation in patients with PE, preventing further progression of the disease with resultant pulmonary infarction^28,29^. These differences could also be explained by the different distribution of age and population^27^.

It is interesting that dyspnea presenting in PE patients, was often accompanied by other symptoms like tachypnea, hypoxia, cough, and hydrothorax. For example, in Cluster 3 of the five clusters generated by cluster analysis, almost all patients in the cluster presented dyspnea and tachypnea, and most presented hypoxia and cough, and some presented hydrothorax. From PCA results, these five symptoms were with the largest loadings in the first principal component. A reasonable explanation was that PE was more likely to be incidentally detected when patients had obvious symptoms^28^. In addition, we recognized that almost half of the PE patients in Cluster 3 were with COPD. COPD patients had a significantly increased risk of dyspnea than those without COPD. The prevalence of dyspnea in PE patients with COPD was 91.3% in this study, which was similar to a latest study^30^. Actually, the patients in Cluster 3 were usually difficult to differentiate with COPD patients. However, if the duration and severity of dyspnea and tachypnea felt different than usual COPD conditions, or the symptoms were not improved after treatment, or the hypoxia was not improved after treatment, the physician should highly suspect the occurrence of PE.

In our study, we also found that in Cluster 1, PE patients with syncope were as the main presentation, accompanied by high oxygen partial pressure (PPO about 96%), and 92.3% of the patients were without edema. The syncope was usually accompanied by hypoxia which was not common in other nervous system diseases. The possible explanation was that embolus sudden blockage in the lung. Such patients should be paid enough attention because of their sudden onset of unknown, lack of typical presentations of PE, and easy to cause misdiagnosed. Therefore, we suggest that patients with this type of presentation should be diagnosed as early as be suspected, and diagnostic performance of PE such as D-dimer testing and CTPA, should be applied for these patients. In Cluster 2 PE patients, there were no typical symptoms. This cluster might be the explanation for under-diagnosis of PE. Possible reason for without typical presentations was that embolus did not fall off to the lungs to formation of PE, or because the patients were on anticoagulant medicine after the formation of lower limb thrombosis. PE patients with cancers and PE patients from obstetrical department usually have no obvious symptoms during PE occurrence and might belong to this cluster. Among PE patients in Cluster 4, the most typical presentation of PE were hemoptysis and cough, accompanied by smoking and COPD. Hemoptysis generally indicated massive PE, but at present PE appeared relatively small probability because of advances in diagnostic techniques. In our study, we found only 11.8% PE patients were with hemoptysis. Researchers recently reported low rates of hemoptysis of PE (only about 5%)^17,18^. The possible reason for the difference might be due to different procedures and level of diagnosis techniques and clinical skills of physicians between our hospital and hospitals from western world. Therefore, we believe that improving the detection and diagnosis of PE, early intervention and treatment would help to reduce the emergence of hemoptysis in patients with PE. Among PE patients in Cluster 5, the most typical presentations of PE were pleuritic pain and cough, accompanied by fever, dyspnea, and tachypnea. Clinicians should pay attention to differentiate such patients with acute myocardial infarction. Pleuritic pain of PE was difficult to describe, and if the pain could not be explained by myocardial infarction or other related diseases, physicians should consider the possibility of PE.

All these findings suggested that more attention should be taken into the under-diagnosis of PE. A timely detection of PE is an essential prerequisite of a prompt effective treatment^31^. The current study indicated that clinical symptoms combined with risk factors might provide useful information in identifying highly susceptible PE patients although clinical manifestations of PE were often nonspecific. The clinical presentation also varies depending on the distribution and size of emboli occluding the pulmonary vasculature, as well as the age and pre-existing co-morbidities of the patients^32,33^. This study identified associated signs and symptoms, clinical risk factors associated with the presentation of PE, which were helpful to aid physicians on the diagnosis of this dangerous and potentially fatal disease. Based on our PCA results, we could establish a scoring system using the loadings of ten symptoms in the largest principal component. However, the overall proportion of the first principal component was only approximately 25%, and the scoring system from this component would not be sensitive and specific enough to diagnose PE patients. However, our study was a good attempt to cluster and clean PE symptoms, and we believe we could develop an accurate scoring system after we accumulate more and more data. As development of artificial intelligence and machine learning techniques, it is possible to deeply study these symptoms and their interactions and combinations and to improve the diagnosis of PE. Moreover in our future study, we aim to analyze all suspicious patients, demonstrate the risk factors of PE incidence, and construct the risk scoring system for PE incidence but not PE patients only.

There were some limitations in this study. The major limitation of our study is its retrospective design. Data collection was based on information available on review of the patient medical records. Second, we did not investigate the etiology of PE in this retrospective study. Third, the clinical findings could be well under-represented due to its dependency on physician assessment and documentation of clinical findings leading to recall bias. Further studies are therefore necessary to validate the diagnostic value of clinical characteristics.

In conclusion, different symptoms were associated with different clinical indicators among PE patients. PE patients could be grouped into different clusters of typical symptoms, which would improve accuracy of diagnosis and prevent adverse events due to delayed diagnosis. The diagnosis of PE remained a challenging task, our results will improve our understanding of clinical symptoms and their potential combinations which are helpful for clinical diagnosis of PE.

## Electronic supplementary material

Supplementary Dataset
