# Supplementary material for: Clinical symptoms and related risk factors in pulmonary embolism patients and cluster analysis based on these symptoms
Source: Sci Rep. 2017 Nov 2;7:14887. doi: 10.1038/s41598-017-14888-7 (PMC5668424; doi:10.1038/s41598-017-14888-7)
Supplement: Supplementary file 1 — Supplementary Dataset [file 41598_2017_14888_MOESM1_ESM.doc]

**Title:** Clinical symptoms and related risk factors in pulmonary embolism patients and cluster analysis based on these symptoms

**Running Title:** Clinical symptoms and pulmonary embolism

**Authors:** Qiao-ying Ji^#1^, Mao-feng Wang^#2^, Cai-min Su^1^, Qiong-fang Yang^1^, Lan-fang Feng^1^, Lan-yan Zhao^1^, Shuang-yan Fang^1^, Fen-hua Zhao^3^, Wei-min Li*^4^

# These authors contributed equally to this work

**Address:** ^1^Department of Respiratory, Affiliated Dongyang Hospital of Wenzhou Medical University, Dongyang, Zhejiang, 322100, China; ^2^Department of Biomedical Sciences Laboratory, Affiliated Dongyang Hospital of Wenzhou Medical University, Dongyang, Zhejiang, 322100, China; ^3^Department of Radiology, Affiliated Dongyang Hospital of Wenzhou Medical University, Dongyang, Zhejiang, 322100, China; ^4^Department of Cardiology, Affiliated Dongyang Hospital of Wenzhou Medical University, Dongyang, Zhejiang, 322100, China

*Correspondence: Professor Wei-min Li, MD. Wuning West Road No. 60, Dongyang, Zhejiang, 322100, China

Tel: 86-579-86856799

Fax: 86-579-86856878

E-mail: dyliwm@126.com

Table 1S. The cluster history for 1-100 clusters from the system clustering analysis

| **Cluster History** | | | | | | | | |
| --- | --- | --- | --- | --- | --- | --- | --- | --- |
| **Number of Clusters** | **Clusters Joined** | | **Freq** | **Semi-partial R-Square** | **R-Square** | **Pseudo F Statistic** | **Pseudo t-Squared** | **Tie** |
| 100 | 350 | 11 | 2 | 0.0009 | 0.969 | 142 | . |  |
| 99 | CL122 | 96 | 6 | 0.0009 | 0.968 | 139 | 6 |  |
| 98 | CL172 | CL379 | 22 | 0.0009 | 0.967 | 137 | . |  |
| 97 | CL143 | 356 | 3 | 0.001 | 0.966 | 135 | 2.6 |  |
| 96 | CL141 | CL213 | 4 | 0.001 | 0.965 | 132 | 5.2 |  |
| 95 | CL156 | CL102 | 5 | 0.001 | 0.964 | 130 | 2.8 |  |
| 94 | CL220 | CL135 | 6 | 0.001 | 0.963 | 128 | 10.8 |  |
| 93 | CL139 | CL154 | 4 | 0.0011 | 0.962 | 126 | 3.3 |  |
| 92 | CL165 | CL169 | 9 | 0.0011 | 0.961 | 124 | . |  |
| 91 | CL218 | CL265 | 12 | 0.0012 | 0.96 | 122 | . |  |
| 90 | CL151 | CL254 | 6 | 0.0012 | 0.958 | 119 | 14.1 |  |
| 89 | CL142 | 102 | 3 | 0.0012 | 0.957 | 117 | 3.3 |  |
| 88 | CL104 | CL281 | 10 | 0.0012 | 0.956 | 116 | 6.6 |  |
| 87 | CL148 | CL144 | 5 | 0.0012 | 0.955 | 114 | 5.2 |  |
| 86 | CL128 | CL93 | 7 | 0.0013 | 0.953 | 112 | 2.8 |  |
| 85 | CL159 | CL132 | 9 | 0.0013 | 0.952 | 111 | 12.7 |  |
| 84 | 290 | CL157 | 3 | 0.0013 | 0.951 | 109 | 4.9 |  |
| 83 | CL120 | CL133 | 4 | 0.0013 | 0.95 | 108 | 2.5 |  |
| 82 | CL125 | 90 | 3 | 0.0013 | 0.948 | 106 | 2.4 |  |
| 81 | CL118 | CL155 | 7 | 0.0013 | 0.947 | 105 | 7.4 |  |
| 80 | CL113 | CL285 | 6 | 0.0014 | 0.946 | 104 | 5.7 |  |
| 79 | CL152 | 234 | 4 | 0.0014 | 0.944 | 103 | 8.2 |  |
| 78 | 512 | CL140 | 3 | 0.0014 | 0.943 | 101 | 3.8 |  |
| 77 | CL84 | CL137 | 7 | 0.0015 | 0.941 | 100 | 3.9 |  |
| 76 | CL115 | CL138 | 8 | 0.0016 | 0.94 | 99 | 8.8 |  |
| 75 | 10 | 94 | 2 | 0.0016 | 0.938 | 97.8 | . |  |
| 74 | CL150 | CL134 | 7 | 0.0016 | 0.937 | 96.6 | 11 |  |
| 73 | CL166 | CL294 | 10 | 0.0016 | 0.935 | 95.6 | . |  |
| 72 | CL107 | CL485 | 7 | 0.0016 | 0.933 | 94.7 | 10.6 |  |
| 71 | CL146 | CL178 | 7 | 0.0017 | 0.932 | 93.7 | 24.4 |  |
| 70 | CL82 | CL100 | 5 | 0.0017 | 0.93 | 92.8 | 1.9 |  |
| 69 | CL186 | CL124 | 9 | 0.0018 | 0.928 | 91.8 | 21.6 |  |
| 68 | CL126 | CL130 | 6 | 0.0018 | 0.927 | 91 | 7.1 |  |
| 67 | 456 | CL105 | 3 | 0.0018 | 0.925 | 90.2 | 2.2 |  |
| 66 | CL121 | CL108 | 7 | 0.0018 | 0.923 | 89.5 | 6.6 |  |
| 65 | CL112 | CL127 | 6 | 0.0018 | 0.921 | 88.8 | 6 |  |
| 64 | CL90 | CL109 | 10 | 0.0019 | 0.919 | 88.1 | 6.5 |  |
| 63 | CL136 | CL263 | 10 | 0.0019 | 0.917 | 87.5 | 38.8 |  |
| 62 | CL94 | CL97 | 9 | 0.002 | 0.915 | 86.8 | 5.2 |  |
| 61 | CL161 | CL83 | 17 | 0.0021 | 0.913 | 86 | 13.8 |  |
| 60 | CL65 | CL110 | 10 | 0.0022 | 0.911 | 85.3 | 4.2 |  |
| 59 | CL76 | CL116 | 14 | 0.0022 | 0.909 | 84.7 | 7.9 |  |
| 58 | CL123 | CL114 | 10 | 0.0023 | 0.907 | 84 | 14.7 |  |
| 57 | CL310 | CL101 | 10 | 0.0023 | 0.904 | 83.4 | 16.2 |  |
| 56 | CL111 | CL241 | 13 | 0.0024 | 0.902 | 82.8 | 24 |  |
| 55 | CL162 | CL167 | 19 | 0.0024 | 0.9 | 82.3 | . |  |
| 54 | CL131 | CL129 | 6 | 0.0024 | 0.897 | 81.8 | 9.9 |  |
| 53 | CL174 | CL261 | 30 | 0.0025 | 0.895 | 81.4 | . |  |
| 52 | CL106 | 422 | 3 | 0.0025 | 0.892 | 81 | 3.2 |  |
| 51 | CL71 | CL87 | 12 | 0.0027 | 0.89 | 80.5 | 6.8 |  |
| 50 | CL96 | CL54 | 10 | 0.0028 | 0.887 | 80.1 | 4.6 |  |
| 49 | CL72 | CL89 | 10 | 0.0029 | 0.884 | 79.6 | 5.8 |  |
| 48 | CL183 | CL119 | 17 | 0.003 | 0.881 | 79.1 | 45.1 |  |
| 47 | CL60 | CL95 | 15 | 0.0031 | 0.878 | 78.7 | 4.8 |  |
| 46 | CL62 | CL81 | 16 | 0.0032 | 0.875 | 78.2 | 6.5 |  |
| 45 | CL80 | CL78 | 9 | 0.0033 | 0.871 | 77.8 | 5.7 |  |
| 44 | CL171 | CL77 | 18 | 0.0035 | 0.868 | 77.3 | 16.6 |  |
| 43 | CL180 | CL246 | 20 | 0.0037 | 0.864 | 76.8 | . |  |
| 42 | CL79 | CL56 | 17 | 0.0037 | 0.86 | 76.4 | 10.8 |  |
| 41 | CL68 | CL75 | 8 | 0.0039 | 0.856 | 76 | 5.3 |  |
| 40 | CL85 | CL52 | 12 | 0.004 | 0.852 | 75.7 | 7.6 |  |
| 39 | CL206 | CL48 | 26 | 0.0041 | 0.848 | 75.3 | 24.4 |  |
| 38 | CL86 | CL57 | 17 | 0.0044 | 0.844 | 74.9 | 9.6 |  |
| 37 | CL182 | CL187 | 28 | 0.0048 | 0.839 | 74.4 | . |  |
| 36 | CL59 | CL73 | 24 | 0.0049 | 0.834 | 74 | 15.1 |  |
| 35 | CL69 | CL39 | 35 | 0.0049 | 0.829 | 73.8 | 15.4 |  |
| 34 | CL74 | CL92 | 16 | 0.005 | 0.824 | 73.5 | 20.4 |  |
| 33 | CL91 | CL40 | 24 | 0.0053 | 0.819 | 73.3 | 11.3 |  |
| 32 | CL38 | CL49 | 27 | 0.0053 | 0.814 | 73.1 | 7.3 |  |
| 31 | CL179 | CL99 | 26 | 0.0054 | 0.808 | 73.1 | 84.9 |  |
| 30 | CL42 | CL103 | 28 | 0.0055 | 0.803 | 73.1 | 14.7 |  |
| 29 | CL55 | CL34 | 35 | 0.0056 | 0.797 | 73.3 | 16.9 |  |
| 28 | CL63 | CL37 | 38 | 0.0056 | 0.792 | 73.6 | 28.9 |  |
| 27 | CL47 | CL66 | 22 | 0.0057 | 0.786 | 74 | 7.7 |  |
| 26 | CL45 | CL67 | 12 | 0.0057 | 0.78 | 74.5 | 5.7 |  |
| 25 | CL44 | CL58 | 28 | 0.0058 | 0.774 | 75.2 | 14.4 |  |
| 24 | CL64 | CL36 | 34 | 0.0068 | 0.768 | 75.7 | 13.5 |  |
| 23 | CL26 | CL70 | 17 | 0.0072 | 0.76 | 76.2 | 5.3 |  |
| 22 | CL27 | CL41 | 30 | 0.0075 | 0.753 | 76.8 | 7.3 |  |
| 21 | CL51 | CL163 | 40 | 0.0086 | 0.744 | 77.1 | 49.2 |  |
| 20 | CL43 | CL46 | 36 | 0.0089 | 0.735 | 77.7 | 21.7 |  |
| 19 | CL160 | CL98 | 48 | 0.0089 | 0.726 | 78.5 | 441 |  |
| 18 | CL88 | CL50 | 20 | 0.0093 | 0.717 | 79.5 | 16.3 |  |
| 17 | CL61 | CL29 | 52 | 0.0095 | 0.708 | 80.8 | 22.8 |  |
| 16 | CL21 | CL25 | 68 | 0.0126 | 0.695 | 81.3 | 26.3 |  |
| 15 | CL53 | CL164 | 68 | 0.0128 | 0.682 | 82.2 | 344 |  |
| 14 | CL32 | CL23 | 44 | 0.0135 | 0.669 | 83.4 | 11.1 |  |
| 13 | CL18 | CL20 | 56 | 0.0139 | 0.655 | 85.1 | 17.6 |  |
| 12 | CL19 | CL30 | 76 | 0.0148 | 0.64 | 87.1 | 43.5 |  |
| 11 | CL28 | CL33 | 62 | 0.0179 | 0.622 | 88.9 | 38 |  |
| 10 | CL12 | CL31 | 102 | 0.0202 | 0.602 | 90.9 | 43.1 |  |
| 9 | CL11 | CL15 | 130 | 0.0213 | 0.581 | 93.8 | 44.4 |  |
| 8 | CL35 | CL22 | 65 | 0.0219 | 0.559 | 98.2 | 26.9 |  |
| 7 | CL24 | CL10 | 136 | 0.0313 | 0.527 | 101 | 46.6 |  |
| 6 | CL17 | CL16 | 120 | 0.0429 | 0.485 | 102 | 67.9 |  |
| 5 | CL9 | CL6 | 250 | 0.0452 | 0.439 | 107 | 56 |  |
| 4 | CL7 | CL8 | 201 | 0.084 | 0.355 | 100 | 86 |  |
| 3 | CL5 | CL13 | 306 | 0.0863 | 0.269 | 101 | 86.9 |  |
| 2 | CL3 | CL14 | 350 | 0.0971 | 0.172 | 114 | 74.6 |  |
| 1 | CL2 | CL4 | 551 | 0.1719 | 0 | . | 114 |  |

Table 2S. Eigenvalues of the correlation matrix from PCA

| **item** | **Eigenvalue** | **Difference** | **Proportion** | **Cumulative** |
| --- | --- | --- | --- | --- |
| 1 | 2.497 | 1.262 | 0.250 | 0.250 |
| 2 | 1.235 | 0.136 | 0.124 | 0.373 |
| 3 | 1.099 | 0.069 | 0.110 | 0.483 |
| 4 | 1.031 | 0.059 | 0.103 | 0.586 |
| 5 | 0.971 | 0.077 | 0.097 | 0.683 |
| 6 | 0.894 | 0.048 | 0.089 | 0.773 |
| 7 | 0.847 | 0.156 | 0.085 | 0.857 |
| 8 | 0.691 | 0.106 | 0.069 | 0.926 |
| 9 | 0.585 | 0.434 | 0.059 | 0.985 |
| 10 | 0.151 |  | 0.015 | 1.000 |
